# Practical synthesis of indoles and benzofurans in water using a heterogeneous bimetallic catalyst

**DOI:** 10.3762/bjoc.9.160

**Published:** 2013-07-16

**Authors:** Cybille Rossy, Eric Fouquet, François-Xavier Felpin

**Affiliations:** 1 Université de Bordeaux, UMR CNRS 5255, ISM, 351 cours de la Libération, 33405 Talence Cedex, France; 2 Université de Nantes, UFR Sciences et Techniques, UMR CNRS 6230, CEISAM, 2 rue de la Houssinière, BP 92208, 44322 Nantes Cedex 3, France

**Keywords:** benzofuran, bimetallic catalyst, heterogeneous catalysis, indole, water

## Abstract

This paper describes the preparation of indoles, azaindoles and benzofurans in pure water by using a new heterogeneous Pd–Cu/C catalyst through a cascade Sonogashira alkynylation–cyclization sequence. Details of the optimization studies and the substrate scope are discussed. This procedure allows the preparation of heterocycles with good yields and is tolerant to a wide variety of functional groups.

## Introduction

**Scheme 1:**
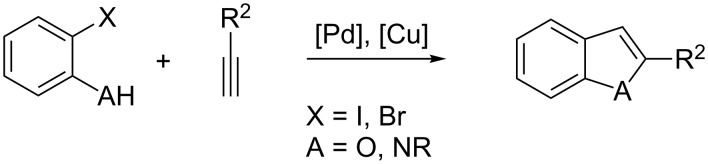
Sonogashira alkynylation–cyclization sequence for indole and benzofuran syntheses.

Heterocycles are ubiquitous building blocks in natural products, bioactive compounds and materials. Amongst the variety of heterocycles, indoles and benzofurans have emerged as privileged structures, especially in medicinal chemistry [1]. This great interest stimulated organic chemists to design efficient and diverse synthetic accesses, abundantly reviewed in the past few years [2–7]. A detailed survey of the recent literature revealed that both indoles and benzofurans can be attained by a cascade Sonogashira alkynylation–cyclization sequence (Scheme 1).

Many efficient catalytic systems have been reported since the pioneering studies of Yamanaka [8] and this approach is now a classic for the synthesis of indole- and benzofuran-containing natural products [9–13] and biologically relevant agents [14–18]. The traditional procedure requires a homogeneous source of palladium and copper in a polar solvent (usually DMF) with an organic base such as Et_3_N. The role of copper is twofold since it acts not only as a cocatalyst for the alkynylation reaction but also as a Lewis acid for the cyclization step. Procedures involving heterogeneous catalysts have been much less explored and essentially limited to the use of heterogeneous Pd catalysts in the presence of CuI [19–21]. Alternatively, copper-free heterogeneous catalysis has been proposed thanks to the Lewis acid properties of zeolite [(NH_4_)Y] used as support [22–24]. A challenging approach consisting of a heterobimetallic catalysis has been occasionally envisaged. This area was pioneered by the group of Dkajovitch, which developed new Pd–Cu/SiO_2_ and Pd–Cu/NaY catalysts for the preparation of the 2-phenylindole in aqueous DMF [25]. More recently, Cu(I)–Pd(II)-containing polymer [26] and palladium–copper on magnetite [27] complexes have been introduced as efficient catalysts for preparing indoles and benzofurans in NMP and toluene, respectively. Considering that a catalyst working in water would be appealing [28], we hypothesized that activated charcoal could be a support of choice for palladium and copper due to its robustness and inertness [29–32]. In this paper we wish to describe the use of a readily available heterobimetallic Pd–Cu/C catalyst for cascade Sonogashira alkynylation–cyclization sequences in pure water leading to indoles, azaindoles and benzofurans.

## Results and Discussion

We recently reported a simple and efficient procedure for preparing a very active, activated-charcoal-supported palladium catalyst for hydrogenation and debenzylation [33]. This homemade Pd/C catalyst was prepared by impregnation of activated charcoal by the reduction of Pd(OAc)_2_ in MeOH under an atmosphere of H_2_ (1 atm). With these previous results in mind, we reasoned that the concomitant impregnation of charcoal with palladium and copper could lead to a heterogeneous bimetallic catalyst, hopefully active for cascade Sonogashira alkynylation–cyclization sequences. Following this aim, we were pleased to find that the stirring of a mixture of Pd(OAc)_2_, Cu(OAc)_2_ and activated charcoal in MeOH under an atmosphere of H_2_ (1 atm) furnished a heterogeneous bimetallic catalyst. The content of Pd (~5 wt %) and Cu (~3.6 wt %) was determined by ICPMS analysis.

With the aim of developing a heterogeneous bimetallic catalysis in water, we took inspiration from the work of Pal et al [34], who reported the preparation of indoles in a Pd/C-catalysed reaction in water with CuI as co-catalyst, Ph_3_P as ligand and 2-aminoethanol as base. To our great pleasure, the application of these conditions with our Pd–Cu/C catalyst, for the coupling of iodoaniline **1** with phenylacetylene (**2**) provided the expected indole **3** with an excellent 90% yield (Table 1, entry 1). Interestingly, Pd-free (Table 1, entry 2) or Cu-free (Table 1, entry 3) conditions were detrimental for the reaction efficiency, suggesting the cooperative role of Pd and Cu. The metal loading was also evaluated and the best compromise was found at 2 mol % Pd (Table 1, entry 4). It is worth noting that phenylacetylene (**2**) was secured from dimerization by O_2_ exclusion through a gentle bubbling of argon. Interestingly, the use of aqueous iPrOH instead of pure water for enhancing the solubility of the organics slightly decreased the efficiency of our catalytic system highlighting the role of water in solvating the substrates.

**Table 1:** Optimization of the catalyst loading.^a^

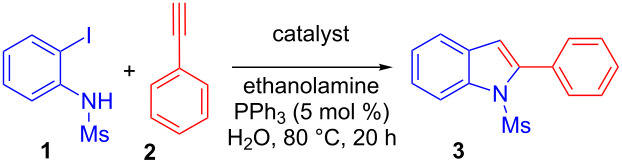				

Entry	Catalyst	Loading Pd (mol %)	Loading Cu (mol %)	Yield (%)^b^

1	Pd–Cu/C	5	6	90
2	Cu/C^c^	–	6	28
3	Pd/C^c^	5	–	65
4	Pd–Cu/C	2	2.4	91
5	Pd–Cu/C	1	1.2	89
6	Pd-Cu/C	0.5	0.6	68

^a^Reaction conditions: 2-iodoaniline (0.5 mmol), phenylacetylene (1 mmol), ethanolamine (1.5 mmol), Ph_3_P (5 mol %) and catalyst (see table) were stirred in water (3 mL) at 80 °C under Ar for 20 h. ^b^Isolated yield. ^c^This catalyst was made following the procedure described for Pd–Cu/C.

**Scheme 2:**
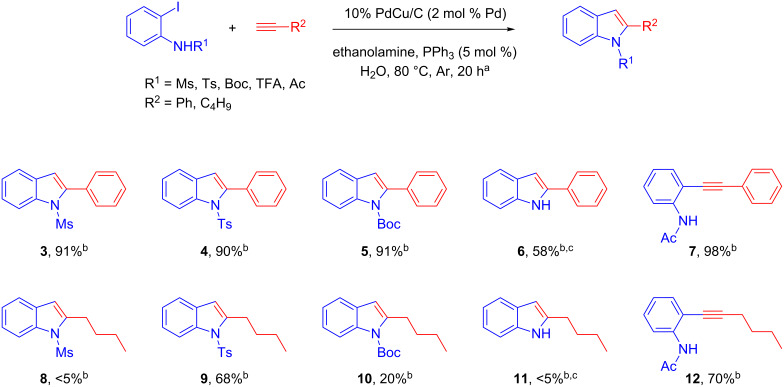
Optimization of the nitrogen protecting group. ^a^Reaction conditions: 2-iodoaniline (0.5 mmol), phenylacetylene (1 mmol), ethanolamine (1.5 mmol), Ph_3_P (5 mol %) and Pd–Cu/C (2 mol % Pd) were stirred in water (3 mL) at 80 °C under Ar for 20 h. ^b^Isolated yield. ^c^The trifluoroacetyl protecting group was used.

We then evaluated the influence of the nitrogen protecting group for the cascade Sonogashira coupling–cyclization sequence on two model reactions (Scheme 2). We observed that the coupling of phenyl acetylene (**2**) with variously N-protected-2-iodoanilines gave contrasted results. Indeed, excellent yields of the corresponding indoles were obtained with anilines bearing mesyl, tosyl or Boc protecting groups (compounds **3**–**5**), while only an uncyclized product was obtained with acetate (compound **7**). The free indole was obtained when a trifluoroacetate function was used as protecting group, albeit in modest yield. The loss of the trifluoroacetate group likely occurred during the workup process. It should be noted that unprotected aniline exclusively led to the uncyclized Sonogashira adduct. Surprisingly a much pronounced N-substituent effect was observed with hexyne as coupling partner, where the tosyl protecting group gave the best result. These results strongly suggest that both electronic and steric factors participate in the cyclization step.

**Scheme 3:**
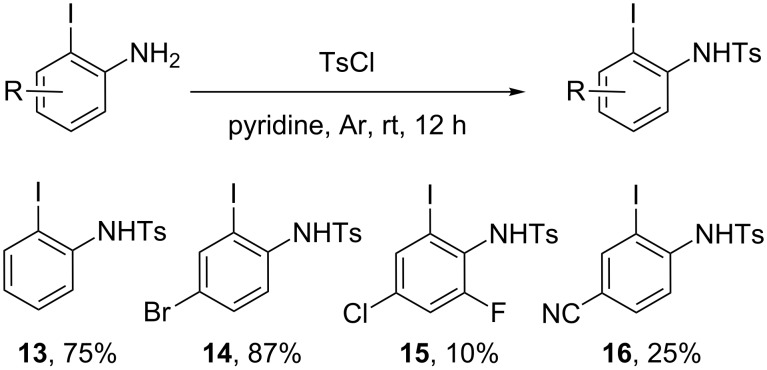
Preparation of N-protected 2-iodoanilines.

**Scheme 4:**
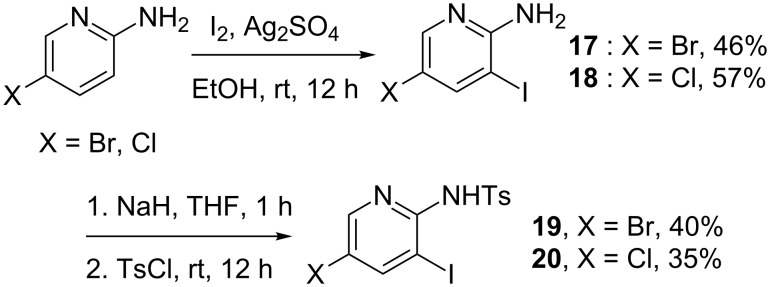
Preparation of N-protected 3-iodo-2-aminopyridines.

With these optimized conditions in hand, we next focused on the reaction scope for which we prepared a series of 2-iodoanilines (Scheme 3) and 2-amino-3-iodopyridines (Scheme 4). 2-Iodoanilines **13–16** were prepared following a standard procedure [35] although only **13** was known at the time of our studies. The preparation of 3-iodo-2-aminopyridines proved to be much more complicated. Indeed, the poor nucleophilicity of amino pyridine **17** and **18** precluded any tosylation under standard conditions. After extensive experimentations we found that the activation of the amino group with a strong base such as NaH allowed the preparation of the expected compounds **19** and **20** in acceptable yields.

**Scheme 5:**
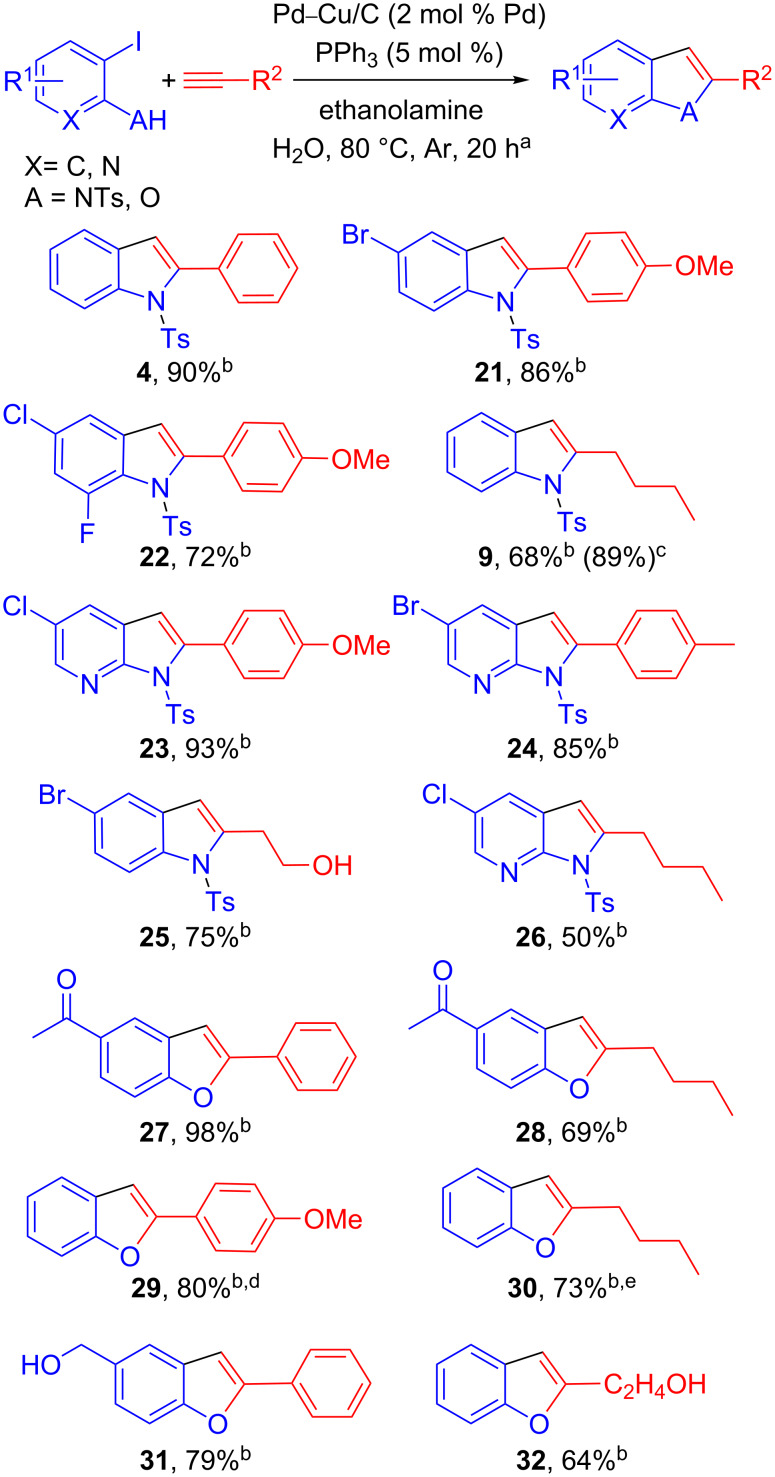
Preparation of indoles, azaindoles and benzofurans. ^a^Reaction conditions: 2-iodoaniline (0.5 mmol), phenylacetylene (1 mmol), ethanolamine (1.5 mmol), Ph_3_P (5 mol %) and Pd–Cu/C (2 mol % Pd) were stirred in water (3 mL) at 80 °C under Ar for 20 h. ^b^Isolated yield. ^c^*N*-Tosyl-2-bromoaniline was used. ^d^Pd–Cu/C (3 mol % Pd) was used. ^e^Pd–Cu/C (5 mol % Pd) was used.

The Sonogashira alkynylation–cyclization sequence was successfully applied to a variety of alkynes and iodo-anilines (Scheme 5). As a general comment, aromatic alkynes consistently furnished a high yield of the corresponding indoles whatever the nature of the electrophile (compounds **4**, **21**, **22**). For instance, the reaction was remarkably selective toward the iodine atom, leaving other halogens (Cl and Br) unaffected. Moreover, 3-iodo-2-aminopyridines **19** and **20** were remarkably reactive, giving the corresponding azaindoles **23** and **24** with excellent yields. On the other hand, aliphatic alkynes gave lower but still synthetically useful yields, even in the presence of halogens and free alcohols. Interestingly, indole **9** was obtained with an improved yield of 89% versus 68% when the corresponding 2-bromoaniline was used instead of the 2-iodoaniline **13**. However, this trend was not observed with aryl acetylenes suggesting a strong halide effect and a sensitivity of the Pd–X bond under our reaction conditions. Indeed, the coupling of aryl acetylenes with bromo-anilines and bromo-phenols showed lower conversion compared to the iodo-congeners. We further evaluated the potential of our Pd–Cu/C catalyst for the alkynylation–cyclization of 2-iodophenols in view of preparing benzofurans. As already observed with the synthesis of indoles, aliphatic alkynes gave slightly lower yields than aryl acetylenes. A remarkable tolerance toward reactive functions such as ketones and alcohols, including benzylic ones, was observed with this catalytic system, highlighting the versatility of our heterogeneous bimetallic catalytic system. This methodology using a homemade Pd–Cu/C catalyst is one of the rare examples of a multitask heterogeneous bimetallic catalysis for tandem or cascade reactions [36–37].

Finally, the recyclability of our homemade Pd–Cu/C catalyst was evaluated for the preparation of indole **4**. Unfortunately, we found that the yield dramatically decreases to 60% (compared to 90% with the fresh catalyst) after the second run and the catalyst was inactive after the third run. ICPMS analyses of the filtered solution after the three runs showed a marginal leaching of Pd (<0.5% based on the amount initially introduced) while a different trend was observed for copper. Indeed, 15% of the copper (based on the initial amount introduced) was detected in the filtered solution after the first run, while virtually no leaching occurred in the subsequent runs. These results suggested that the diminished activity cannot be attributed to the copper leaching. A similar deactivation was observed by Djakovitch and co-workers with their Pd–Cu/NaY catalyst that likely occurred during the separation from the reaction media [23]. We believe that a similar behaviour can explain the catalyst deactivation observed in our studies. The use of flow technology might address this issue as stated by Djakovitch et al., and we are currently investigating this promising alternative.

## Conclusion

In summary we have described a new water-compatible heterogeneous Pd–Cu/C catalyst for cascade Sonogashira alkynylation–cyclization sequences leading to indoles, azaindoles and benzofurans. The procedure allows the preparation of useful heterocycles under water-only conditions and with inexpensive ligand and base. Current studies focusing on the preparation of more robust and recyclable heterogeneous bimetallic catalysts for batch and flow processes are underway in our laboratory.

## Experimental

Catalyst preparation: The Pd–Cu/C was prepared by the following procedure. Pd(OAc)_2_ (119 mg, 0.5 mmol), Cu(OAc)_2_ (170 mg, 0.9 mmol) and charcoal (1 g) were dispersed in MeOH (100 mL). Then, hydrogen gas was bubbled through the solution for 5 minutes to remove oxygen. The resulting mixture was stirred for 12 h at 25 °C under H_2_ (1 atm, balloon). The catalyst was filtered under a Millipore membrane (filters nylon 0.45 µm, 47 mm), washed with MeOH and dried under vacuum. ICP analyses were performed on the filtrate to verify the final Pd-metal loading on carbon to be 5 wt % and Cu-metal loading on carbon to be 3.6 wt %.

General procedure for the preparation heterocycles: In a sealed tube, aryl iodide (0.5 mmol, 1.0 equiv), PPh_3_ (5 mol %) and catalyst Pd–Cu/C (2 mol %) were suspended in H_2_O (3 mL) previously degassed. Then, acetylene derivative (1.0 mmol, 2.0 equiv) and ethanolamine (1.5 mmol, 3.0 equiv) were added. The resulting mixture was stirred 20 h at 80 °C under argon atmosphere. After cooling to room temperature, DCM (10 mL) and H_2_O (10 mL) were added and the mixture was filtered over a pad of Celite^®^. The aqueous layer was extracted twice with DCM (2 × 10 mL). The collected organic extracts were washed by brine (20 mL), dried over MgSO_4_, filtered and concentrated under reduced pressure.

## Supporting Information

File 1 Experimental details and characterization of compounds synthesized.
