# Supplementary material for: Practical synthesis of indoles and benzofurans in water using a heterogeneous bimetallic catalyst
Source: Beilstein J Org Chem. 2013 Jul 16;9:1426–31. doi: 10.3762/bjoc.9.160 (PMC3740569; doi:10.3762/bjoc.9.160)
Supplement: File 1 — Experimental details and characterization of compounds synthesized. [file Beilstein_J_Org_Chem-09-1426-s001.pdf]

Supporting Information

for

Practical synthesis of indoles and benzofurans in water using a heterogeneous bimetallic catalyst

Cybille Rossy[1,2], Eric Fouquet[1], François-Xavier Felpin*[,2]

Address: [1]Université de Bordeaux, UMR CNRS 5255, ISM, 351 cours de la Libération, 33405 Talence Cedex, France and [2]Université de Nantes, UFR Sciences et Techniques, UMR CNRS 6230, CEISAM, 2 rue de la Houssinière, BP 92208, 44322 Nantes Cedex 3, France.

Email: François-Xavier Felpin - fx.felpin@univ-nantes.fr

*Corresponding author

Experimental details and characterization of compounds synthesized

1. General procedure

1.1. Apparatus

All commercial materials were used without further purification, unless indicated. ^{1}H NMR and ^{13}C NMR were recorded on Bruker DPX-200 FT (^{1}H: 200 MHz, ^{13}C: 50.2 MHz), Bruker Avance 300 FT (^{1}H: 300 MHz, ^{13}C: 75.3 MHz), Bruker DPX-400 FT (^{1}H: 400 MHz, ^{13}C: 100.2 MHz). Chemical shifts from proton and carbon NMR spectra are reported in ppm relative to the $CDCl_3$ peak at 7.26 ppm (^{1}H) or 77.0 ppm (^{13}C). Coupling constants *J* are reported in hertz (Hz). The following abbreviations are used for the multiplicities: s, singlet; d, doublet; t, triplet; q, quartet; qt, quintet; st, sextet; m, multiplet; br, broad; dd, doublet of

doublet. Infrared (IR) spectra were recorded as neat samples on NaCl plates or as KBr pellets. Melting points were not corrected and were determined by using a Stuart Scientific apparatus (SMP3). Yields refer to isolated material determined to be pure by NMR spectroscopy and thin-layer chromatography (TLC), unless specified otherwise in the text. Analytical TLC was performed on Fluka Silica Gel 60 F_{254}.

1.2. Materials

All commercial materials were used without further purification, unless indicated. $Pd(OAc)_2$ (45.9–48.4% of metal) and $Cu(OAc)_2$ were obtained from Alfa Aesar, methanol Chromasolv® for HPLC > 99.9% and activated charcoal Darco® G-60 (100 mesh) were obtained from Sigma–Aldrich.

2. Preparation of Pd–Cu/C

The Pd–Cu/C was prepared by the following procedure. $Pd(OAc)_2$ (119 mg, 0.5 mmol), $Cu(OAc)_2$ (170 mg, 0.9 mmol) and charcoal (1 g) were dispersed in MeOH (100 mL). Then, the hydrogen gas was bubbled through the solution for 5 minutes to remove the oxygen. The resulting mixture was stirred for 12 h at 25 °C under H_2 (1 atm, balloon). The catalyst was filtered under Millipore membrane (filters nylon 0.45 µm, 47 mm), washed with MeOH and dried under vacuum. ICP analyses were performed on the filtrate to verify the final Pd-metal loading on carbon to be 5 wt % and Cu-metal loading on carbon to be 3.6 wt %.

3. Cross-coupling reactions

3.1 Synthesis of N-protected-2-iodoanilines.

***N*-(2-Iodophenyl)methanesulfonamide (1)** [1]. 2-Iodoaniline (5.00 g, 24.4 mmol) and DMAP (447.1 mg, 3.66 mmol) were suspended in pyridine (25 mL) and cooled to 0–5 °C. Then, methanesulfonyl chloride (2.4 mL, 31.72 mmol) was added drop by drop and the resulting mixture was heated under reflux for 12 h. After completion of the reaction, the mixture was cooled to 5–10 °C and neutralized with HCl (0.2 M). The aqueous phase was extracted with EtOAc (3 × 50 mL). The collected organic extracts were washed with brine (2 × 100 mL), dried on $MgSO_4$, filtered and concentrated under reduced pressure. Purification

1. Layek, M.; Lakshmi, U.; Kalita, D.; Barange, D. K.; Islam, A.; Mukkanti K.; Pal. M. *Beilstein J. Org. Chem.* **2009**, *5*, No. 46.

by flash chromatography (30% EtOAc-petroleum ether) gave **1** (6.57 g, 91%) as a brown solid. mp 94 °C (lit.[2] 93–94 °C). IR (KBr) ν 3283, 3074, 1582 cm^{-1}. ^{1}H NMR ($CDCl_3$, 300 MHz) δ 3.01 (s, 3H), 6.64 (br, 1H), 6.88–6.99 (m, 1H), 7.27–7.39 (m, 1H), 7.63 (dd, 1H, *J* = 1.4, 8.0 Hz), 7.82 (dd, 1H, *J* = 1.4, 8.0 Hz). ^{13}C NMR ($CDCl_3$, 75 MHz) δ 40.9, 93.0, 123.3, 128.1, 130.7, 138.7, 140.2. HRMS (ESI) calcd for $C_7H_8NO_2NaSI$ (M + Na^+) 319.9212, found 319.9217.

3.2 General procedure for tosylation of 2-iodoanilines.

To a solution of 2-iodoaniline (9 mmol) in anhydrous pyridine (20 mL) was added in *p*-toluenesulfonyl chloride (10.8 mmol). The reaction mixture was stirred overnight at room temperature under an argon atmosphere then quenched with water (20 mL). The solution was extracted with DCM (3 × 100 mL) and the combined organic extracts were washed with 10% aqueous $CuSO_4$ (2 × 50 mL), brine (2 × 100 mL), dried on $MgSO_4$, filtered and concentrated under reduced pressure.

***N*-(2-Iodophenyl)-4-methylbenzenesulfonamide (13).** Purification by flash chromatography (20% EtOAc-petroleum ether) gave **13** (2.5 g, 75%) as a white powder. mp 93 °C (lit.[3] 90–92 °C). IR (KBr) ν 3064, 2922, 1593, 1469 cm^{-1}. ^{1}H NMR ($CDCl_3$, 300 MHz) δ 2.35 (s, 3H), 6.77–6.83 (m, 2H), 7.19 (d, 2H, *J* = 7.2 Hz), 7.25–7.30 (m, 1H), 7.62 (m, 4H). ^{13}C NMR ($CDCl_3$, 75 MHz) δ 21.7, 122.6, 127.0, 127.6 (2C), 129.6, 129.8 (2C), 136.1, 137.7, 139.2, 144.4. HRMS (ESI) calcd for $C_{13}H_{12}NO_2NaSI$ (M + Na^+) 395.9525, found 395.9535.

***N*-(4-Bromo-2-iodophenyl)-4-methylbenzenesulfonamide (14).** The experiment was carried out on a 1.7 mmol scale of 2-iodo-4-bromoaniline. Purification by flash chromatography (30% EtOAc-petroleum ether) gave **14** (660 mg, 87%) as a white solid. mp 121 °C. IR (KBr) ν 3051, 2928, 1593, 1330 cm^{-1}. ^{1}H NMR ($CDCl_3$, 300 MHz) δ 2.40 (s, 3H), 7.24 (d, 2H, *J* = 8.0 Hz), 7.42 (dd, 1H, *J* = 2.2, 8.7 Hz), 7.54 (d, 1H, *J* = 8.7 Hz), 7.62 (d, 2H, *J* = 8.4 Hz). ^{13}C NMR ($CDCl_3$, 75 MHz) δ 21.8, 118.9, 123.5, 127.6 (2C), 129.9 (2C), 132.7, 135.8, 137.0, 141.0, 144.7. HRMS (FD) calcd for $C_{13}H_{12}BrNO_2S$ (M) 324.9772, found 324.9771.

***N*-(4-Chloro-2-fluoro-6-iodophenyl)-4-methylbenzenesulfonamide (15).** The experiment was carried out on a 1.1 mmol scale of 2-fluoro-3-chloro-6-iodoaniline. Purification by flash

2. Sakamoto, T.; Yoshinori, K.; Shigeki, I.; Tatsuo, N.; Yamanaka. H. *Chem. Pharm. Bull.* **1988**, *36*, 1305-1308.
3. Mailyan, A. K.; Geraskin, I. M.; Nemykin, V. N.; Zhdankin. V. V. *J. Org. Chem.*, **2009**, *74*, 8444–8447.

chromatography (10% EtOAc-petroleum ether) gave **15** (76 mg, 10%) as a white solid. mp 142–144 °C. IR (KBr) ν 3254, 3074, 1559, 1336 cm^{-1}. ^{1}H NMR ($CDCl_3$, 300 MHz) δ 2.44 (s, 3H), 6.30 (br, 1H), 7.12 (dd, 1H, *J* = 2.3, 9 Hz), 7.29 (d, 2H, *J* = 8.0 Hz), 7.60 (s, 1H), 7.69 (d, 2H, *J* = 8.3 Hz). ^{13}C NMR ($CDCl_3$, 75 MHz) δ 21.8, 100.1, 117.8, 118.1, 127.7 (2C), 129.8 (2C), 134.7, 137.3, 144.5, 155.7, 159.2. HRMS (FD) calcd for $C_{13}H_{10}ClFINO_2S$ (M) 424.9150, found 424.9136.

***N*-(4-Cyano-2-iodophenyl)-4-methylbenzenesulfonamide (16).** The experiment was carried out on a 1.0 mmol scale of 2-iodo-4-cyanoaniline. Purification by flash chromatography (10% EtOAc-petroleum ether) gave **16** (99 mg, 25%) as a yellow oil. IR (neat) ν 3025, 2358, 1669 cm^{-1}. ^{1}H NMR ($CDCl_3$, 300 MHz) δ 2.36 (s, 3H), 6.95 (d, 1H, *J* = 7.9 Hz), 7.38–7.45 (m, 2H), 7.63 (s, 1H), 7.75 (d, 2H, *J* = 8.1 Hz). ^{13}C NMR ($CDCl_3$, 75 MHz) δ 21.6, 84.7, 104.3, 119.3, 119.8, 128.6 (2C), 129.6 (2C), 131.7, 136.4, 137.2, 141.9, 159.2. HRMS (FD) calcd for $C_{14}H_{11}IN_2O_2S$ (M) 397.9167, found 397.9129.

3.3 General procedure for the synthesis of 3-iodo-2-aminopyridines.

Iodine (9.9 mmol) was added to a mixture of 2-aminopyridine (7.6 mmol) and silver sulphate (9.9 mmol) in ethanol (50 mL). The resulting mixture was stirred overnight at room temperature. The mixture was filtered under pad of Celite® and washed with ethanol, and the filtrate was concentrated under reduced pressure. Then, the residue was dissolved in EtOAc (50 mL), and the solution was washed with a saturated aqueous Na_2SO_3 solution (100 mL). The aqueous phase was extracted with EtOAc (3 × 100 mL). The collected organic extracts were dried on Na_2SO_4, filtered and concentrated under reduced pressure.

5-Bromo-3-iodopyridin-2-amine (17). Purification by flash chromatography (20% EtOAc-pentane) gave **17** (1.02 g, 46%) as a brown powder. mp 105–109 °C. IR (KBr) ν 3447, 3268, 3151, 1619 cm^{-1}. ^{1}H NMR ($CDCl_3$, 300 MHz) δ 4.97 (br, 2H), 7.95 (dd, 1H, *J* = 2.1 Hz), 8.05 (dd, 1H, *J* = 2.1 Hz). ^{13}C NMR ($CDCl_3$, 75 MHz) δ 77.8, 107.3, 148.3, 148.5, 156.6. HRMS (ESI) calcd for $C_5H_5N_2BrI$ (M + H^+) 298.8675, found 298.8667.

5-Chloro-3-iodopyridin-2-amine (18). Purification by flash chromatography (20% EtOAc-pentane) gave **18** (1.1 g, 57%) as a brown powder. mp 93–97 °C. IR (KBr) ν 3450, 3291, 3140, 2904, 1635 cm^{-1}. ^{1}H NMR ($CDCl_3$, 400 MHz) δ 5.07 (br, 1H), 7.82 (dd, 1H, *J* =

1.8 Hz), 7.97 (dd, 1H, *J* = 1.8 Hz). ^{13}C NMR ($CDCl_3$, 100 MHz) δ 120.3, 145.4, 146.4, 156.4 HRMS (ESI) calcd for $C_5H_5N_2ClI$ (M + H^+) 254.9180, found 254.9189.

3.4. General procedure for tosylation of 3-iodo-2-aminopyridines.

To a suspension of NaH (60% in oil, 3.0 mmol) in dry THF (10 mL) was added dropwise a solution of 3-iodo-2-aminopyridine (2.0 mmol) in dry THF (15 mL) at 0 °C and stirred at room temperature for 1 h. Then, to the mixture was added a solution of *p*-toluenesulfonyl chloride (3.0 mmol) in dry THF (10 mL) at 0 °C. The resulting mixture was allowed to warm up to room temperature overnight. Then, the mixture was acidified by 2 M aqueous HCl solution, extracted with EtOAc (2 × 40 mL), dried over Na_2SO_4, filtered and concentrated under reduced pressure.

***N*-(5-Bromo-3-iodopyridin-2-yl)-4-methylbenzenesulfonamide (19).** Purification by flash chromatography (10% EtOAc-petroleum ether) gave **19** (361 mg, 40%) as a brown solid. mp 85 °C. IR (KBr) ν 3255, 3064, 2922, 1565, 1436, 1338, 1160 cm^{-1}. ^{1}H NMR ($CDCl_3$, 300 MHz) δ 2.41 (s, 3H), 5.03 (br s, 1H), 7.29 (d, 2H, *J* = 8.2 Hz), 7.95 (s, 1H), 7.99 (d, 2H, *J* = 8.16 Hz), 8.05 (d, 2H, *J* = 8.2 Hz), 8.20 (s, 1H). ^{13}C NMR ($CDCl_3$, 75 MHz) δ 21.8, 107.3, 128.8 (2C), 129.5 (2C), 136.4, 144.6, 148.4, 148.5, 149.0, 156.5. HRMS (FD) calcd for $C_{12}H_{10}BrIN_2O_2S$ (M) 451.8691, found 451.8706.

***N*-(5-Chloro-3-iodopyridin-2-yl)-4-methylbenzenesulfonamide (20).** Purification by flash chromatography (10% EtOAc-petroleum ether) gave **20** (479 mg, 59%) as a white solid. mp 128–130 °C. IR (KBr) ν 3255, 3064, 2922, 1565, 1436, 1338, 1160 cm^{-1}. ^{1}H NMR ($CDCl_3$, 300 MHz) δ 2.42 (s, 3H), 7.30 (d, 2H, *J* = 8.4 Hz), 7.55 (br, 1H), 7.93 (s, 1H), 8.01 (d, 2H, *J* = 8.3 Hz), 8.13 (s, 1H). ^{13}C NMR ($CDCl_3$, 75 MHz) δ 21.8, 80.5, 125.8, 128.8 (2C), 129.5 (2C), 136.4, 144.6, 146.3, 146.6, 148.9. HRMS (ESI) calcd for $C_{12}H_{10}N_2O_2NaSClI$ (M + Na^+) 430.9088, found 430.9103.

3.5. General procedure for the preparation of indoles.

In a sealed tube, aryl iodide (0.5 mmol, 1.0 equiv), PPh_3 (5 mol %) and catalyst Pd–Cu/C (2 mol %) were suspended in previously degassed H_2O (3 mL). Then, acetylene derivative (1.0 mmol, 2.0 equiv) and ethanolamine (1.5 mmol, 3.0 equiv) were added. The resulting mixture was stirred 20 h at 80 °C under an argon atmosphere. After cooling to room temperature, DCM (10 mL) and H_2O (10 mL) were added, and the mixture was filtered over a

pad of Celite®. The aqueous layer was extracted twice with DCM (2 × 10 mL). The collected organic extracts were washed by brine (20 mL), dried on $MgSO_4$, filtered and concentrated under reduced pressure.

2-Phenyl-1*H*-indole (6). Purification by flash chromatography (10% EtOAc-petroleum ether) gave **6** (49 mg, 58 %) as a light brown solid. mp 190 °C (lit.[4] 191–192 °C). ^{1}H NMR ($CDCl_3$, 300 MHz) δ 6.83 (s, 1H), 7.09–7.27 (m, 2H), 7.31–7.47 (m, 4H), 7.62–7.89 (m, 3H), 8.33 (br, 1H). ^{13}C NMR ($CDCl_3$, 75 MHz) δ 100.6, 111.1, 119.9, 121.6, 122.4, 125.4, 1291 (2C), 130.3 (2C), 130.6, 133.4, 137.2, 138.7. The ^{1}H and ^{13}C NMR data correspond to those reported in the literature [5].

2-Butyl-1*H*-indole (11). Purification by flash chromatography (10% EtOAc-petroleum ether) gave **11** (3.5 mg, 4%) as a white solid. ^{1}H NMR ($CDCl_3$, 400 MHz) δ 0.91 (t, 3H, J = 7.3 Hz), 1.39 (st, 2H, J = 7.2 Hz), 1.76 (qt, 2H, J = 7.3 Hz), 2.85 (t, 2H, J = 7.5 Hz), 6.30 (s, 1H), 7.15–7.25 (m, 2H), 7.37–7.45 (m, 2H), 8.36 (br, 1H). ^{13}C NMR ($CDCl_3$, 100 MHz) δ 12.9, 21.9, 29.6, 30.6, 100.9, 114.7, 117.6, 123.8, 127.8, 128.5, 130.0, 143.1. The ^{1}H and ^{13}C NMR data correspond to those reported in the literature [5].

1-(Methylsulfonyl)-2-phenyl-1*H*-indole (3). Purification by flash chromatography (10% EtOAc-petroleum ether) gave **3** (121.9 mg, 91 %) as a brown solid. mp 103 °C (lit.[6] 103–105 °C). IR (KBr) ν 3012, 2929, 1451, 1360, 1175 cm^{-1}. ^{1}H NMR ($CDCl_3$, 300 MHz) δ 2.74 (s, 3H), 6.72 (s, 1H), 7.35–7.40 (m, 2H), 7.42–7.46 (m, 3H), 7.56–7.62 (m, 3H), 8.13 (d, 1H, J = 8.73 Hz). ^{13}C NMR ($CDCl_3$, 75 MHz) δ 39.6, 113.2, 116.0, 121.1, 124.7, 125.2, 127.8 (2C), 129.0, 130.3, 130.4, 132.1, 138.1, 142.1. HRMS (ESI) calcd for $C_{15}H_{13}NO_2NaS$ (M + Na$^+$) 294.0559, found 294.0572.

2-Phenyl-1-tosyl-1*H*-indole (4). Purification by flash chromatography (5% EtOAc-petroleum ether) gave **4** (156 mg, 90%) as a brown solid. mp 146 °C (lit.[7] 145–147 °C). IR (KBr) ν 3065, 1595, 1367 cm^{-1}. ^{1}H NMR ($CDCl_3$, 300 MHz) δ 2.17 (s, 3H), 6.4 (s, 1H), 6.92 (d, 2H, J = 8.0 Hz), 7.28–7.31 (m, 4H), 7.34–7.44 (m, 6H), 8.20 (d, 1H, J = 8.3 Hz). ^{13}C NMR ($CDCl_3$, 75 MHz) δ 21.7, 113.7, 116.8, 120.8, 124.4, 124.9, 127.0 (2C), 127.6 (2C), 128.8,

---

4. Boyer, A.; Isono N.; Lackner, S.; Lautens. M *Tetrahedron*, **2010**, *66*, 6468-6482.
5. Djakovitch, L.; Dufaud, V.; Zaidi, R. *Adv. Synth. Catal*. **2006**, 348, 715-724.
6. Boyer, A.; Isono N.; Lackner, S.; Lautens. M *Tetrahedron*, **2010**, *66*, 6488-6482.
7. Palimkar, S. S.; Kumar, P. H.; Lahoti R. J.; Srinivasan. K. V. *Tetrahedron*, **2006**, *62*, 5109-5115.

129.3 (2C), 130.5 (2C), 130.7, 132.6, 134.9, 138.4, 142.3, 144.6. HRMS (FD) calcd for $C_{21}H_{17}NO_2S$ (M) 347.0980, found 347.0970.

***tert*-Butyl 2-phenyl-1*H*-indole-1-carboxylate (5).** The experiment was carried out on a 0.4 mmol scale of *N*-(*tert*-butoxycarbonyl)-2-iodoaniline. Purification by flash chromatography (1% EtOAc-petroleum ether) gave **5** (106 mg, 91%) as a yellow solid. mp 77 °C (lit.[8] 76–77 °C). IR (KBr) ν 3013, 1732, 1579, 1220 cm^{-1}. ^{1}H NMR ($CDCl_3$, 300 MHz) δ 1.56 (s, 9H), 6.90 (td, 1H, *J* = 1.1, 7.6 Hz), 7.21–7.33 (m, 5H), 7.38 (dd, 1H, *J* = 1.4, 7.7 Hz), 7.43–7.49 (m, 2H), 8.09 (d, 1H, *J* = 8.4 Hz). ^{13}C NMR ($CDCl_3$, 75 MHz) δ 28.4, 80.9, 84.7, 96.2, 111.3, 117.7, 122.2, 122.8, 128.6, 128.8 (2C), 129.7, 131.6 (2C), 131.8, 139.6, 152.5. HRMS (EI) calcd for $C_{19}H_{19}NO_2$ (M) 293.1416, found 293.1405.

***N*-(2-(Phenylethynyl)phenyl)acetamide (7).** Purification by flash chromatography (10% EtOAc-petroleum ether) gave **7** (115 mg, 98%) as a white solid. mp 119 °C (lit.[9] 118–120 °C). IR (KBr) ν 3305, 3030, 2772, 1661, 1578, 1255 cm^{-1}. ^{1}H NMR ($CDCl_3$, 300 MHz) δ 2.25 (s, 3H), 6.97 (t, 1H, *J* = 6.7 Hz), 7.27–7.34 (m, 4H), 7.40–7.50 (m, 3H), 7.90 (br, 1H), 8.33 (d, 1H, *J* = 8.3 Hz). ^{13}C NMR ($CDCl_3$, 75 MHz) δ 25.1, 84.4, 96.5, 111.9, 119.5, 122.5, 123.5, 128.7, 129.1, 129.9, 131.6, 131.7, 139.1, 168.3. HRMS (ESI) calcd for $C_{16}H_{13}NONa$ ($M + Na^+$) 258.0889, found 258.0884.

2-Butyl-1-(methylsulfonyl)-1*H*-indole (8). Purification by flash chromatography (10% EtOAc-petroleum ether) gave **8** (5 mg, 4%) as a brown solid. mp 80 °C (lit.[10] 79–80 °C). IR (KBr) ν 3019, 2956, 2936, 1592, 1323 cm^{-1}. ^{1}H NMR ($CDCl_3$, 300 MHz) δ 0.97 (t, 3H, *J* = 7.4 Hz), 1.46 (st, 2H, *J* = 7.4 Hz), 2.95 (t, 2H, *J* = 7.9 Hz), 2.99 (s, 3H), 6.46 (d, 1H, *J* = 0.9 Hz), 7.25 (m, 2H), 7.47 (m, 1H), 7.99 (m, 1H). ^{13}C NMR ($CDCl_3$, 75 MHz) δ 14.1, 22.6, 28.7, 31.2, 40.5, 108.6, 114.3, 120.4, 123.8, 124.1, 130.0, 136.9, 142.6. HRMS (EI) calcd for $C_{13}H_{17}NO_2S$ (M) 251.0980, found 251.0970.

2-Butyl-1-tosyl-1*H*-indole (9). Purification by flash chromatography (10% EtOAc-petroleum ether) gave **9** (111.2 mg, 68%) as a yellow solid. mp 87 °C (lit.[11] 88–90 °C). IR (KBr)

8. R'kyek, O.; Halland, N.; Lindenschmidt, A.; Alonso, J.; Lindemann, P.; Urmann M.; Nazaré. M. *Chem. Eur. J.* **2010**, *16*, 9986-9989.
9. Saito, T.; Ogawa, S.; Takei, N.; Kutsumura N.; Otani. T. *Org. Lett.* **2011**, *13*, 1098-1101.
10. Isono N.; Lautens. M. *Org. Lett.* **2009**, *11*, 1329-1331.
11. Djakovitch, L.; Dufaud, V.; Zaidi. R. *Adv. Synth. Catal.* **2006**, *348*, 715-724.

ν 2955, 1593, 1365, 1145 cm^{-1}. ^{1}H NMR ($CDCl_3$, 400 MHz) δ 0.88 (t, 3H, *J* = 7.4 Hz), 1.37 (st, 2H, *J* = 7.4 Hz), 1.65 (qt, 2H, *J* = 7.4 Hz), 2.25 (s, 3H), 2.91 (t, 2H, *J* = 7.6 Hz), 6.30 (s, 1H), 7.01–7.18 (m, 5H), 7.32 (d, 1H, *J* = 7.4 Hz), 7.53 (d, 2H, *J* = 8.3 Hz), 8.09 (d, 1H, *J* = 8.2 Hz). ^{13}C NMR ($CDCl_3$, 100 MHz) δ 14.1, 21.7, 22.6, 28.9, 31.1, 108.7, 114.9, 120.2, 123.6, 123.9, 126.4, 129.9 (2C), 130.0 (2C), 136.4, 137.4, 142.7, 144.7. HRMS (ESI) calcd for $C_{19}H_{21}NO_2NaS$ (M + Na^+) 350.1185, found 350.1188.

***tert*-Butyl 2-butyl-1*H*-indole-1-carboxylate (10)**. The experiment was carried out on a 0.4 mmol scale of *N*-(*tert*-butoxycarbonyl)-2-iodoaniline. Purification by flash chromatography (1% EtOAc-petroleum ether) gave **10** (22 mg, 20%) as a colourless oil. IR (neat) ν 3013, 1732, 1579, 1220 cm^{-1}. ^{1}H NMR ($CDCl_3$, 300 MHz) δ 0.99 (t, 3H, *J* = 7.4 Hz), 1.46 (st, 2H, *J* = 7.4 Hz), 1.63–1.78 (m, 2H), 1.71 (s, 3H), 3.50 (t, 2H, *J* = 7.8 Hz), 6.40 (s, 1H), 7.16–7.24 (m, 2H), 7.43 (d, 1H, *J* = 7.2 Hz), 8.07 (d, 1H, *J* = 8.2 Hz). ^{13}C NMR ($CDCl_3$, 75 MHz) δ 14.6, 22.8, 28.3, 28.9, 31.4, 84.9, 101.6, 117.0, 119.8, 120.3, 124.9, 128.1, 132.0, 137.4, 150.6. The ^{1}H and ^{13}C NMR data correspond to those reported in the literature [12].

***N*-(2-(Hex-1-ynyl)phenyl)acetamide (12).** In a sealed tube, *N*-(2-iodophenyl)acetamide (130.5 mg, 0.5 mmol), PPh_3 (6.6 mg, 5 mol%) and catalyst Pd–Cu/C (21.7 mg, 2 mol % Pd) were suspended in H_2O (3 mL) previously degassed with argon. Then, hexyne (0.12 mL, 1.0 mmol) and ethanolamine (91 µL, 1.5 mmol) were added. The resulting mixture was stirred 20 h at 80 °C under an argon atmosphere. After cooling to room temperature, DCM (10 mL) and H_2O (10 mL) were added, and the mixture was filtered over a pad of Celite®. The aqueous layer was extracted twice with DCM (2 × 10 mL). The collected organic extracts were washed by brine (20 mL), dried on $MgSO_4$, filtered and concentrated under reduced pressure. Purification by flash chromatography (10% EtOAc-petroleum ether) gave **12** (75.4 mg, 70%) as a yellow solid. mp 46 °C (lit.[13] 46–47 °C). IR (KBr) ν 300, 2227, 1662, 755 cm^{-1}. ^{1}H NMR ($CDCl_3$, 300 MHz) δ 0.99 (t, 3H, *J* = 7.3 Hz), 1.48–1.56 (m, 2H), 1.58–1.67 (m, 2H), 2.21 (s, 3H), 2.52 (t, 2H, *J* = 6.8 Hz), 6.99 (t, 1H, *J* = 7.6 Hz), 7.27–7.30 (m, 1H), 7.31–7.37 (m, 1H), 7.94 (br, 1H), 8.37 (d, 1H, *J* = 8.3 Hz). ^{13}C NMR ($CDCl_3$, 75 MHz) δ 13.6, 19.3, 22.1, 24.9, 30.8, 76.1, 97.9, 112.6, 119.0, 123.3, 128.9, 131.6, 139.0, 168.2. HRMS (ESI) calcd for $C_{14}H_{17}NONa$ (M + Na^+) 238.1202, found 238.1208.

---

12. Kuwano R.; Kashiwabara. M. *Org. Lett*. **2006**, 8, 2653-2655.
13. Rudisill, D. E.; Stille. J. K. *J. Org. Chem*. **1989**, 54, 5856-5866.

5-Bromo-2-(4-methoxyphenyl)-1-tosyl-1*H*-indole (21). Purification by flash chromatography (20% EtOAc-petroleum ether) gave **21** (196 mg, 86%) as a yellow oil. IR (neat) *ν* 2930, 2835, 1602, 1504, 1376 cm^{-1}. ^1H NMR ($CDCl_3$, 300 MHz) δ 2.29 (s, 3H), 3.88 (s, 3H), 6.45 (s, 1H), 6.88 (dd, 2H, *J* = 1.9, 7.0 Hz), 6.97 (dd, 2H, *J* = 1.9, 6.9 Hz), 7.04 (d, 2H, *J* = 8.2 Hz), 7.45–7.51 (m, 3H), 7.56–7.58 (m, 1H), 8.27 (d, 1H, *J* = 8.7 Hz). ^{13}C NMR ($CDCl_3$, 75 MHz) δ 21.6, 55.4, 114.1, 115.5 (2C), 116.8, 119.6, 123.7, 124.5, 126.9 (2C), 128.0, 129.4 (2C), 130.8, 133.1 (2C), 134.7, 137.7, 143.1, 144.8, 159.7. HRMS (ESI) calcd for $C_{22}H_{18}BrNO_3SNa$ (M + Na^+) 478.0265, found 478.0286.

5-Chloro-7-fluoro-2-(4-methoxyphenyl)-1-tosyl-1*H*-indole (22). The experiment was carried out on a 0.1 mmol scale of *N*-(4-chloro-2-fluoro-6-iodophenyl)-4-methylbenzenesulfonamide. Purification by flash chromatography (20% EtOAc-petroleum ether) gave **22** (196 mg, 72%) as a colourless oil. IR (neat) *ν* 2947, 2863, 1634, 1559, 1328, 1270 cm^{-1}. ^1H NMR ($CDCl_3$, 300 MHz) δ 2.47 (s, 3H), 3.85 (s, 3H), 6.86 (s, 1H), 7.11–7.25 (m, 3H), 7.34 (d, 2H, *J* = 8.2 Hz), 7.46 (d, 2H, *J* = 8.9 Hz), 7.71 (s, 1H), 7.92 (d, 2H, *J* = 8.4 Hz). ^{13}C NMR ($CDCl_3$, 75 MHz) δ 22.3, 59.7, 102.3, 107.4, 115.8, 117.4 (2C), 121.9, 124.3, 125.9 (2C), 128.1, 128.6 (2C), 128.9, 131.6 (2C), 134.4, 139.8, 150.6, 160.3. HRMS (FD) calcd for $C_{22}H_{17}ClFNO_3S$ (M) 429.8462, found 429.8451.

3.6. General procedure for the preparation of azaindoles and benzofurans.

In a sealed tube, aryl iodide (0.5 mmol, 1.0 equiv), PPh_3 (5 mol %) and catalyst Pd–Cu/C (2 mol % Pd) were suspended in H_2O (3 mL) previously degassed with argon. Then, the acetylene derivative (1.0 mmol, 2.0 equiv) and ethanolamine (1.5 mmol, 3 equiv) were added. The resulting mixture was stirred for 20 h at 80 °C under an argon atmosphere. After cooling to room temperature, DCM or EtOAc (10 mL) and H_2O (10 mL) were added and the mixture was filtered over a pad of Celite®. The aqueous layer was extracted twice with DCM or EtOAc (2 × 10 mL). The collected organic extracts were washed with brine (20 mL), dried on $MgSO_4$, filtered and concentrated under reduced pressure.

5-Chloro-2-(4-methoxyphenyl)-1-tosyl-1*H*-7-azaindole (23). The experiment was carried out on a 0.25 mmol scale of *N*-(5-chloro-3-iodopyridin-2-yl)-4-methylbenzenesulfonamide. Purification by flash chromatography (10% EtOAc-petroleum ether) gave **23** (96 mg, 93%) as a yellow powder. mp 158–160 °C. IR (KBr) *ν* 3064, 1610, 1375, 1286 cm^{-1}. ^1H NMR

($CDCl_3$, 300 MHz) δ 2.33 (s, 3H), 3.88 (s, 3H), 6.38 (s, 1H), 6.98 (d, 2H, *J* = 8.8 Hz), 7.16 (d, 2H, *J* = 8.0 Hz), 7.68–7.71 (m, 3H), 8.37 (s, 2H). ^{13}C NMR ($CDCl_3$, 75 MHz) δ 21.7, 55.4, 107.8, 113.3 (2C), 123.5, 124.5, 127.8 (2C), 127.9, 129.5 (2C), 131.3 (2C), 135.5, 142.9, 144.2, 145.1, 148.2, 160.5. HRMS (FD) calcd for $C_{21}H_{17}N_2O_3SNaCl$ (M + Na^+) 435.0540, found 435.0555. Anal. calcd. for $C_{21}H_{17}ClN_2O_3S$: C, 61.17; H, 4.13; N, 6.80; S, 7.78; found C, 60.96; H, 4.24; N, 6.22; S, 7.00.

5-Bromo-2-(4-methoxyphenyl)-1-tosyl-1*H*-7-azaindole (24). The experiment was carried out on a 0.25 mmol scale of *N*-(5-bromo-3-iodopyridin-2-yl)-4-methylbenzenesulfonamide. Purification by flash chromatography (10% EtOAc-petroleum ether) gave **24** (93.7 mg, 85%) as a yellow oil. IR (neat) *ν* 2922, 2853, 1694, 1380 cm^{-1}. ^1H NMR ($CDCl_3$, 600 MHz) δ 2.34 (s, 3H), 2.45 (s, 3H), 6.40 (s, 1H), 7.18 (d, 2H, *J* = 8.1 Hz), 7.27 (d, 2H, *J* = 7.4 Hz), 7.43 (d, 2H, *J* = 8.1 Hz), 7.74 (d, 2H, *J* = 8.4 Hz), 7.88 (d, 1H, *J* = 2.2 Hz), 8.47 (d, 1H, *J* = 2.2 Hz). ^{13}C NMR ($CDCl_3$, 150 MHz) δ 21.6, 21.8, 107.8, 115.9, 124.1, 127.9 (2C), 128.6 (2C), 129.4, 129.5 (2C), 129.9 (2C), 130.9, 135.6, 139.4, 144.2, 145.1, 145.2, 148.4. HRMS (ESI) calcd for $C_{21}H_{17}N_2O_2NaSBr$ (M + Na^+) 463.0086, found 463.0074.

5-Bromo-2-(2-hydroxy)ethanol-1-tosyl-1*H*-indole (25). Purification by flash chromatography (30% EtOAc-petroleum ether) gave **25** (65.1 mg, 75%) as a yellow oil. IR (KBr) *ν* 3293, 3081, 2978, 1590, 1372 cm^{-1}. ^1H NMR ($CDCl_3$, 300 MHz) δ 1.89 (br, 1H), 2.33 (s, 3H), 3.26 (t, 2H, *J* = 6.2 Hz), 3.99 (t, 2H, *J* = 6.2 Hz), 6.43 (s, 1H), 7.19 (d, 2H, *J* = 8.5 Hz), 7.35 (dd, 1H, *J* = 1.9, 8.5 Hz), 7.53 (d, 1H, *J* = 1.9 Hz), 7.58 (d, 2H, *J* = 8.3 Hz), 8.02 (d, 1H, *J* = 8.9 Hz). ^{13}C NMR ($CDCl_3$, 75 MHz) δ 21.7, 32.6, 61.7, 109.7, 116.4, 117.2, 123.0, 126.2, 126.3, 126.4 (2C), 127.1, 130.1 (2C), 131.5, 135.7, 136.0, 139.8, 145.3. HRMS (FD) calcd for $C_{17}H_{16}BrNO_3S$ (M-H^+) 393.0042, found 393.0042.

2-Butyl-5-chloro-1-tosyl-1*H*-7-azaindole (26). Purification by flash chromatography (20% EtOAc-petroleum ether) gave **26** (90 mg, 50%) as a colourless oil. IR (neat) *ν* 2958, 2930, 1596, 1550, 1385, 1191 cm^{-1}. ^1H NMR ($CDCl_3$, 300 MHz) δ 0.99 (t, 2H, *J* = 7.3 Hz), 1.42–1.55 (m, 2H,), 1.72–1.82 (m, 2H), 2.36 (s, 3H), 3.12 (t, 2H, *J* = 7.3 Hz), 6.26 (s, 1H), 7.3 (d, 2H, *J* = 8.6 Hz), 7.64 (d, 1H, *J* = 2.3 Hz), 7.97 (d, 2H, *J* = 8.4 Hz), 8.26 (d, 1H, *J* = 2.3 Hz). ^{13}C NMR ($CDCl_3$, 75 MHz) δ 14.0, 21.7, 22.6, 29.3, 30.9, 103.9, 122.9, 127.2 (2C), 127.8,

129.7 (2C), 136.3, 142.0, 145.2, 145.7, 147.3. HRMS (FD) calcd for $C_{18}H_{19}ClN_2O_2S$ (M) 362.0856, found 362.0859.

2-Phenyl-5-ethanoylbenzofuran (27). Purification by flash chromatography (20% EtOAc-petroleum ether) gave **27** (107.9 mg, 98%) as a yellow solid. mp 160 °C. IR (KBr) ν 3090, 2918, 1673, 1566, 1354 cm^{-1}. ^1H NMR ($CDCl_3$, 300 MHz) δ 2.66 (s, 3H), 7.09 (s, 1H), 7.39–7.50 (m, 3H), 7.56 (d, 1H, *J* = 8.6 Hz), 7.86–7.89 (m, 2H), 7.96 (dd, 1H, *J* = 1.8, 8.6 Hz), 8.24 (s, 1H). ^{13}C NMR ($CDCl_3$, 75 MHz) δ 26.9, 101.8, 111.3, 122.3, 125.2 (2C), 125.3, 129.0 (2C), 129.2, 130.0, 133.1, 157.7, 197.8. HRMS (FD) calcd for $C_{16}H_{12}O_2$ (M) 236.0837, found 236.0835.

2-Butyl-5-ethanoylbenzofuran (28). Purification by flash chromatography (10% EtOAc-petroleum ether) gave **28** (73.1 mg, 69%) as a yellow oil. IR (neat) ν 2959, 2932, 1681, 1601, 1360, 1269 cm^{-1}. ^1H NMR ($CDCl_3$, 300 MHz) δ 0.96 (t, 3H, *J* = 7.3 Hz), 1.36–1.46 (m, 2H), 1.68–1.79 (m, 2H), 2.64 (s, 3H), 2.78 (t, 2H, *J* = 7.4 Hz), 6.44 (s, 1H), 7.42 (d, 1H, *J* = 8.6 Hz), 7.87 (dd, 1H, *J* = 1.8, 8.6 Hz), 8.12 (d, 1H, *J* = 1.7 Hz). ^{13}C NMR ($CDCl_3$, 75 MHz) δ 13.8, 22.3, 26.8, 28.2, 29.6, 102.4, 110.7, 121.5, 124.1, 129.2, 132.4, 157.4, 161.6, 197.9. HRMS (FD) calcd for $C_{14}H_{16}O_2$ (M) 216.1150, found 216.1152.

2-(4-Methoxyphenyl)benzofuran (29). The experiment was carried out on a 0.5 mmol scale of 2-iodophenol with Pd–Cu/C (3 mol % Pd). Purification by flash chromatography (10% EtOAc-petroleum ether) gave **29** (79.3 mg, 80%) as a white solid. mp 150 °C (lit.[14] 150–152 °C). IR (KBr) ν 3120, 3052, 2960, 1610, 1504, 1250 cm^{-1}. ^1H NMR ($CDCl_3$, 300 MHz) δ 3.87 (s, 3H), 6.89 (s, 1H), 6.98 (dd, 2H, *J* = 2.1, 6.9 Hz), 7.27–7.21 (m, 2H), 7.49–7.57 (m, 2H), 7.80 (dd, 2H, *J* = 2.1, 6.8 Hz). ^{13}C NMR ($CDCl_3$, 75 MHz) δ 55.5, 99.8, 111.1, 114.3, 114.4 (2C), 120.7, 122.9, 123.5, 123.9, 126.6 (2C), 129.6, 154.8, 156.2, 160.1. HRMS (ESI) calcd for $C_{15}H_{13}O_2$ (M + H^+) 225.0910, found 225.0909.

2-Butylbenzofuran (30). Purification by flash chromatography (5% EtOAc-petroleum ether) gave **30** (63.8 mg, 73%) as a colourless oil. IR (neat) ν 2958, 2932, 2872, 1725 cm^{-1}. ^1H NMR ($CDCl_3$, 300 MHz) δ 0.96 (t, 3H, *J* = 7.6 Hz), 1.38–1.54 (m, 2H), 1.69–1.76 (m, 2H), 2.26 (t, 2H, *J* = 6.8 Hz), 2.77 (t, 2H, *J* = 7.3 Hz), 6.37 (s, 1H), 7.17–7.20 (m, 2H), 7.39–

14. Csékei, M.; Novak, Z.; Kotschy. A. *Tetrahedron*, **2008**, *64*, 8992-8996.

7.42 (m, 1H), 7.46–7.49 (m, 1H). ^{13}C NMR ($CDCl_3$, 75 MHz) δ 13.9, 19.0, 22.0, 30.5, 101.9, 110.8, 120.3, 122.5, 123.1, 159.9. MS (EI) *m*/*z* 174.16 (M^+). The ^{1}H and ^{13}C NMR data corresponds to that reported in literature [15].

2-Phenyl-5-hydroxymethylbenzofuran (31). Purification by flash chromatography (20% EtOAc-petroleum ether) gave **31** (88.6 mg, 79%) as a white solid. mp 141.8 °C. IR (KBr) *ν* 3313, 3051, 2858, 1561 cm^{-1}. ^{1}H NMR ($CDCl_3$, 400 MHz) δ 4.81 (s, 2H), 6.96 (s, 1H), 7.01 (s, 1H), 7.24 (d, 1H, *J* = 8.7 Hz), 7.36 (t, 1H, *J* = 7.4 Hz), 7.46 (t, 2H, *J* = 7.4 Hz), 7.56 (d, 2H, *J* = 7.9 Hz), 7.86–7.88 (m, 2H). ^{13}C NMR ($CDCl_3$, 100 MHz) δ 65.8, 101.3, 110.0, 121.0, 122.4, 125.1 (2C), 128.7, 128.9 (2C), 130.6, 137.7, 155.3, 156.5. HRMS (ESI) calcd for $C_{15}H_{13}O_2$ ($M + H^+$) 225.0910, found 225.0909.

2-(2-Hydroxyethyl)benzofuran (32). Purification by flash chromatography (40% EtOAc-petroleum ether) gave **32** (52.1 mg, 64%) as a yellow oil. IR (neat) *ν* 3248, 2985, 2851, 1630, 1520 cm^{-1}. ^{1}H NMR ($CDCl_3$, 300 MHz) 2.95 (td, 2H, *J* = 0.5, 6.2 Hz), 3.89 (t, 2H, *J* = 6.2 Hz), 6.42 (s, 1H), 7.09–7.18 (m, 2H), 7.42–7.45 (m, 1H), 7.49–7.52 (m, 1H). ^{13}C NMR ($CDCl_3$, 75 MHz) δ 32.1, 60.7, 103.7, 110.9, 120.5, 122.7, 123.6, 128.8, 154.9, 156.1. HRMS (ESI) calcd for $C_{10}H_{10}O_2$ (M) 162.0681, found 162.0672.

15. Liang, Y.; Tang, S.; Zhang, X.-D.; Mao, L.-Q.; Xie Y.-X.; Li, J.-H. *Org. Lett.* **2006**, *8*, 3017-3020.
